# Hepatic steatosis in patients with HIV-Hepatitis C Virus coinfection: is it associated with antiretroviral therapy and more advanced hepatic fibrosis?

**DOI:** 10.1186/1756-0500-1-46

**Published:** 2008-07-15

**Authors:** Sumita Verma, Robert D Goldin, Janice Main

**Affiliations:** 1 Hepatology Section, Department of Medicine, Imperial College at St Mary's Hospital, London, UK; 2 Department of Cellular Pathology, Imperial College at St Mary's Hospital, London, UK

## Abstract

**Background and aims:**

Patients with HIV and hepatitis C virus (HCV) coinfection are at increased risk of developing hepatic steatosis. The aims of this study were to assess the prevalence of steatosis in a cohort with HIV-HCV coinfection, and to determine an association, if any, between steatosis, antiretroviral therapy (ART), and advanced hepatic fibrosis.

**Patients and methods:**

HIV-HCV coinfected patients were retrospectively identified from the HIV clinic. ART was classified as none, nucleoside reverse transcriptase inhibitors (NRTIs) only, highly active antiretroviral therapy (HAART) only, and sequential therapy (initial NRTIs followed by HAART). Fibrosis stage and necroinflammation grade were assessed by the modified HAI (Ishak) scoring method. Steatosis was graded as 0–3.

**Results:**

Sixty patients were identified. The overall prevalence of hepatic steatosis was 58%. Those that received HAART only had a lower prevalence of steatosis (41%) compared to those on NRTIs only (70%) or sequential therapy (82%). Independent predictors of hepatic steatosis were absence of HAART only therapy, OR 2.9, p = 0.09, and presence of cirrhosis, OR 4.6, p = 0.044. Forty five percent of the patients had advanced fibrosis (fibrosis stage ≥ 3). NI grade (OR 1.9, p = 0.030), and steatosis grade (OR 3.6, p = 0.045), were independent predictors of advanced fibrosis.

**Conclusion:**

Hepatic steatosis is associated with more advanced hepatic fibrosis in the HIV-HCV coinfected population. HAART only therapy (rather than NRTIs only or sequential therapy) appears to be associated with a lower prevalence of hepatic steatosis. This may be one of the mechanisms by which HAART could attenuate hepatic fibrosis in such a cohort.

## Introduction

Highly active antiretroviral therapy (HAART) has significantly improved survival in patients with human immunodeficiency virus (HIV) infection [1]. Increasing attention is now being focused on co infection with other viruses like hepatitis C (HCV). Because of similar routes of transmission, approximately 25–30% of patients with HIV are also coinfected with HCV [2]. Factors associated with more advanced hepatic fibrosis in HCV infection include HIV coinfection [3] and hepatic steatosis, (prevalence of 47%–79%) [4-6].

Patients with HIV are also at increased risk of developing hepatic steatosis due to multiple factors including antiretroviral therapy (ART), obesity, hyperglycemia, lipodystrophy, and coinfection with HCV [2,7-11]. In HIV-HCV coinfection prevalence of hepatic steatosis varies between 40–72.1% [9-13]. In the coinfected population, the association between ART and steatosis, and whether steatosis is associated with advanced fibrosis remains controversial [9-11]. The aims of this study were therefore to assess whether in those with HIV-HCV coinfection

1. Use of ART is associated with hepatic steatosis

2. Hepatic steatosis is an independent predictor of advanced hepatic fibrosis

3. Hepatic steatosis is associated with fibrosis progression in serial liver biopsies

## Patients and methods

The study period was from 1990–2005. Patients with HIV-HCV coinfection were identified from the Pathology and HIV database after which, their charts and computerised chemical pathology and histology databases reviewed. To be included in the study the patients had to be

1. HCV antibody and or HCV PCR (qualitative) positive

2. HIV antibody positive

3. Have had a liver biopsy.

The indications for a liver biopsy in most patients were abnormal liver tests. All the liver biopsies had been reviewed by RG who was blinded to the clinical information. The fibrosis stage (0–6) and necroinflammatory (NI) grade (0–18) were assessed by the modified HAI (Ishak) scoring system [14]. Steatosis was graded, (depending on % of hepatocytes containing fat), into grade 0 (< 5%), grade 1 (<33%), grade 2 (33%–66%), and grade 3 (>66%).

HCV disease duration and fibrosis progression were calculated as before [15]. Lipoatrophy was stated to be present if mentioned in the patient records. Diabetes mellitus (DM) was defined by presence of one or more of the following: fasting blood glucose > 7 mmol/l, being on anti diabetic medications, and/or a note in the patient record stating that there was a history of DM.

Anti-HCV antibody testing, HCV RNA and HIV RNA quantification and HCV genotyping were performed in the hospital virology laboratory using standard commercial kits (Abbott, Bayer, Roche).

Alcohol abuse was defined as either consumption of > 3 units of alcohol/day (approximately 40 gms/day), and/or a written note in patient records stating that there was a history of alcohol excess. Details of ART were also recorded to assess duration of therapy prior to the liver biopsy. ART was classified as [16].

1. None

2. NRTIs only

3. HAART only. Those whose HIV was diagnosed in or after 1996 and were therefore treated with HAART at onset. For the purpose of this study HAART was defined as use of any two nucleoside reverse transcriptase inhibitors (NRTIs) with any protease inhibitor (PI) or non nucleoside reverse transcriptase inhibitors (NNRTI)

4. Sequential therapy: Those whose HIV was diagnosed prior to 1996; hence were initially treated with NRTIs and then converted to HAART after 1996.

Ethnicity was classified (according to the HIV database) into White, Other European and Others.

The following patients were excluded

1. Presence of liver disease of another etiology, e.g. active hepatitis B infection, autoimmune liver disease, genetic hemochromatosis, and cholestatic liver disease

2. Medical records/clinical data not available

### Statistical Analysis

Data are presented as mean ± SD except the HIV and HCV viral load (VL) which are presented as median (range). Differences between continuous variables were compared using the Mann Whitney (U) Test and between categorical variables by the Chi Square and Fischer's Exact Test. Binary logistic regression analysis was performed to assess independent predictors of hepatic steatosis and fibrosis stage ≥ 3. The statistical software programme used was SPSS version 14 (Chicago, IL)

## Results

From 1990–2005 there were sixty-six patients with HIV who had been coded as having had a liver biopsy for HCV. Six were excluded, and therefore 60 patients were found suitable for the study. Of these sixty patients 52 (87%) had abnormal transaminases. All were HCV antibody positive, and all but one was HCV RNA (qualitative) positive. Eight patients had undergone more than one biopsy, of whom seven had had two and one three biopsies.

Table 1 shows data in the whole cohort at the time of the most recent biopsy. The mean age at HIV diagnosis was 33.3 ± 8.1 years. Mean duration of HIV prior to biopsy was 6.2 ± 5.5 years. At the time of the biopsy, 53 (88%) had a CD4 count ≥ 200 and 52% had undetectable HIV VL. Overall, 12 had received an NNRTI, 9 a PI and 12 had received both. As regards NRTI, 17 had received stavudine (d4T), 13 didanosine (ddI) and 33 zidovudine (AZT). Of the 17 who had received no ART at the time of the biopsy, 16 were long-term non-progressors.

**Table 1:** Data in whole cohort (n = 60) at the time of the most recent liver biopsy

Age at biopsy (yrs)	39.4 ± 8.2
Ethnicity	
White	24 (40%)
Other European	24 (40%)
Others	12 (20%)
Male	54 (90%)
Weight (kg)	72.3 ± 10.1
> 75 kg	24 (40%)
Diabetes Mellitus	3 (5%)
Lipoatrophy	5 (8%)
Risk factors for HCV	
IDU	32 (53%)
Blood transfusion	3 (5%)
Homosexual	19 (32%)
Heterosexual	3 (5%)
Tattoos/fisting	3 (5%)
Unknown	1 (2%)
Alcohol Abuse	18 (30%)
CD4+ (/mm^3^)	450 (100–1200)
HIV VL (copies/ml)	UD (UD-210,955)
**Antiretroviral therapy**	
None	17 (28%)
NRTI only	10 (17%)
HAART only	22 (37%)
Sequential therapy	11 (18%)
HCV VL (IU/ml)*	869,299 (2716–14,975,540)
HCV genotype 1*	30/42 (71%)
HCV disease duration (yrs)	16.4 ± 8.0
Liver Biopsy**	
Fibrosis stage	2.8 ± 1.7
Fibrosis progression rate/yr	0.21 ± 0.16
(FPR)*	
Cirrhosis	15 (25%)
Necroinflammatory grade	3.7 ± 1.7
Hepatic steatosis	35 (58%)

*HCV viral load and genotyping unavailable in 16 and 18 patients respectively

*FPR unavailable in 22 patients

** Data in median (range): fibrosis stage 2 (0–6), necroinflammatory grade 3 (1–9), fibrosis progression rate/yr 0.15 (0–0.67), steatosis grade 1 (0–3)

UD: undetectable

Ethnicity: In Other European the country of origin was as follows: Italy n = 8, Portugal n = 8, Spain n = 5, Germany n = 1, France n = 1 and Venezuela n = 1. Others included Brazil n = 3, Russia n = 3, Yugoslavia n = 3, India n = 1, Iran n = 1 and Libya n = 1

**Figure 1:**
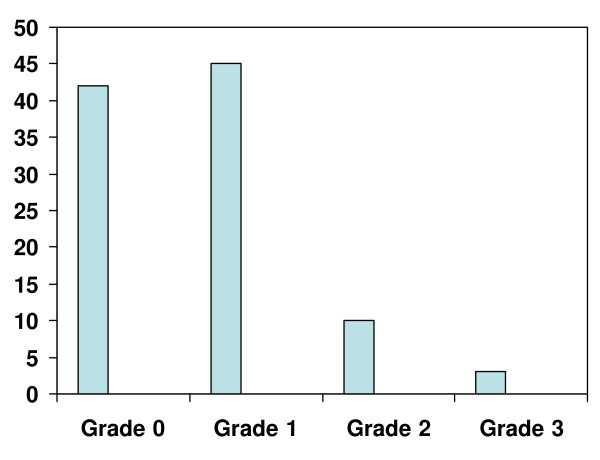
The prevalence of hepatic steatosis (in percentage) in HIV-HCV coinfected Patients.

**Figure 2:**
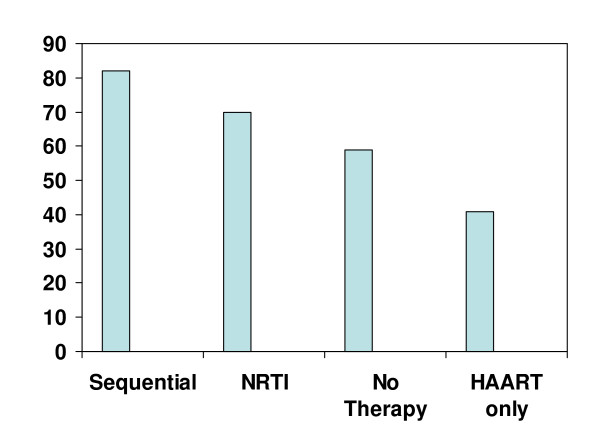
**Prevalence of hepatic steatosis (in percentage) depending on type of therapy received**. Number of patients with steatosis in the 4 groups was as follows: Sequential 9/11, NRTI 7/10, No therapy 10/17 and HAART only 9/22. HAART only vs NRTIs only, p = 0.11. HAART only vs sequential therapy, p = 0.064.

Some degree of hepatic steatosis was present in 58% of the patients, with the majority having grade 1 steatosis (Fig 1). Table 2 shows data in those with and without steatosis. Those who received NRTIs only (70%) or sequential therapy (82%) were more likely to have hepatic steatosis compared to those that received HAART only (41%), p = 0.11 and 0.64 respectively), (Fig 2). The use of HAART only therapy was protective against development of steatosis (Table 2). The mean steatosis grade in the HAART only group was 0.5 ± 0.8. This was lower than that seen in the NRTI only (1.2 ± 1.0, p = 0.20), sequential therapy group (0.9 ± 0.5, p = 0.63), and no therapy group (0.6 ± 0.6). Further sub group analysis indicated the prevalence of steatosis as follow: stavudine (70%), didanosine (61%), zidovudine (63%), any PI therapy (62%), and any NNRTI therapy (58%).

**Table 2:** Data in patients with and without hepatic steatosis

	Hepatic steatosis	Hepatic steatosis	p value
	Yes	No	

	n = 35	n = 25	

Age at biopsy (yrs)	39.0 ± 8.9	40.0 ± 7.2	ns
Age at HIV diagnosis (yrs)	32.9 ± 8.9	34.0 ± 6.8	ns
Male	32 (91%)	22 (88%)	ns
ethnicity	14 (40%)	9 (36%)	ns
Weight (kg)	74.2 ± 9.2	69.7 ± 10.8	0.74
Weight > 75 kg	17 (48%)	7 (28%)	ns
Diabetes mellitus	2 (6%)	1 (4%)	ns
Alcohol	11 (31%)	7 (28%)	ns
Antiretroviral therapy			
No therapy	10 (28%)	7 (28%)	ns
NRTI	7 (20%)	3 (12%)	ns
Duration (mths)	22.3 ± 18.8	21.7 ± 12.4	ns
HAART only	9 (26%)	13 (52%)	0.037
Duration (mths)	30.4 ± 27.3	29.5 ± 27.1	ns
Sequential	9 (26%)	2 (8%)	0.08
Duration (mths)	92.8 ± 37.2	106.0	ns
Received PI	13 (37%)	8 (32%)	ns
Received NNRTI	14 (40%)	10 (40%)	ns
Received stavudine	12 (34%)	5 (20%)	ns
Received didanosine	8 (23%)	5 (20%)	ns
Received zidovudine	21 (60%)	12 (48%)	ns
Time from HIV diagnosis to HAART (mths)	47.4 + 38.7	40.4 + 50.9	ns
CD4 count (/mm3)	410 (110–1100)	490 (100–1200)	ns
HIV VL (copies/ml)	UD (UD-210955)	70 (UD-121843)	ns
HCV VL (IU/ml)	441309 (2716–769231)	1286755 (29451–14975540)	ns
Non 1 genotype	6/21 (28%)	6/21 (28%)	ns
Cholestrol	4.3 ± 1.2	4.1 ± 1.08	ns
HDL	1.1 ± 0.5	1.1 ± 0.3	ns
Triglyceride	2.2 ± 2.0	1.5 ± 0.6	ns
Cholestrol/HDL ratio	4.8 ± 2.2	3.8 ± 1.0	ns
Fasting blood glucose	6.1 + 6.4	5.1 ± 0.9	ns
ALT (IU/L)	131 + 149.8	104 ± 74.6	ns
Fibrosis stage	3.0 ± 1.9	2.5 ± 1.7	ns
NI grade	3.7 ± 1.9	3.7 + 1.7	ns
Cirrhosis	12 (34%)	3 (12%)	0.049

Normal range for lab values: ALT <40 iu/L, Bilirubin <17 umol/L, Alkaline phosphates 30–130 iu/L, Albumin 35–41 g/L, Cholestrol <5 mmol/L, triglycerides <2.30 mmol/L, HDL cholesterol >1.00 mmol/L, Cholestrol/HDL <5.00 mmol/L, fasting blood glucose 3–5.5 mmol/L

HDL: high density lipoprotein

UD: undetectable

Data on lipids unavailable in 10 patients

There was no difference in durations of different forms of ART in those with and without steatosis (Table 2). The overall duration of ART was longer in those with sequential therapy (94.3 ± 35.5 mths), compared to NRTI only (22.9 ± 17.3 mths) and HAART only groups (29.9 ± 26.5 mths), p < 0.05. However presence of steatosis was not associated with duration of ART (50.6 ± 43.0 vs. 32.7 ± 30.6 mths). Duration of stavudine (32.8 ± 21.7 vs. 45.8 ± 25.4), and didanosine (24.6 ± 16.6 vs. 31.2 ± 29.7) were also no different in those with and without steatosis (p value = 1.0 and 0.80 respectively). All factors that were significantly associated with steatosis on univariate analysis (p ≤ 0.1) (HAART only therapy, sequential therapy, and presence of cirrhosis) were then entered into a stepwise logistic regression model. Independent predictors of hepatic steatosis were absence of HAART only therapy, OR 2.9 (95% CI 0.84–9.9), p = 0.09 and presence of cirrhosis, OR 4.6 (95% CI 1.0–20.4), p = 0.044. Because of the small sample size we could not analyze the association between diabetes mellitus (n = 3), genotype 3 (n = 6), lipoatrophy (n = 5) and hepatic steatosis.

Table 3 shows data in those with ≥ vs. < stage 3 fibrosis. There were no significant differences in serum bilirubin, transaminases and HCV VL (data not shown).

**Table 3:** Data in patients with fibrosis stage ≥ vs. < 3

	Fibrosis stage ≥ 3 n = 27	Fibrosis stage < 3 n = 33	p value
Age at biopsy (yrs)	40.6 ± 8.7	38.3 ± 7.8	ns
Age exposed to HCV (yrs)	20.3 ± 11.0	24.4 ± 7.2	ns
Age at HIV diagnosis (yrs)	33.6 ± 9.6	33.1 ± 6.7	ns
HIV diagnosed in or after 1996	11 (41%)	23 (70%)	0.023
White	8 (30%)	15 (45%)	ns
Alcohol	6 (22%)	12 (36%)	ns
Diabetes Mellitus	1 (4%)	2 (6%)	ns
Weight	70.7 ± 10.0	73.5 ± 10.2	ns
Weight ≥ 75 Kg	11 (41%)	13 (39%)	
Cholestrol	4.5 ± 1.3	4.0 ± 1.0	ns
Triglycerides (TG)	2.1 ± 1.8	1.8 ± 1.5	ns
Cholestrol/TG ratio	5.0 ± 2.2	3.8 ± 1.3	0.02
Platelet count (/mm^3^)	163 ± 65.5	228 ± 66.8	0.001
**Therapy received**			
None	5 (18%)	12 (36%)	Ns
NRTI	4 (15%)	6 (18%)	Ns
Duration	11.2 ± 8.4	29.3 ± 17.1	0.16
Only HAART	11 (41%)	11 (33%)	Ns
Duration	29.5 ± 26.5	30.4 ± 27.9	Ns
Sequential	7 (26%)	4 (12%)	Ns
Duration	105 ± 23.3	77.5 ± 47.3	0.39
Steatosis	18 (67%)	17 (51%)	Ns
Steatosis grade	1.0 ± 0.9	0.5 ± 0.5	0.028
Necroinflammatory grade	4.4 ± 1.9	3.1 ± 1.3	0.007
CD4 count (/mm^3^)	370 (130–1100)	510 (100–1200)	0.06


**HIV VL (copies/ml)**	UD (UD-210955)	28.5 (UD-59540)	Ns

UD: undetectable

**Figure 3:**
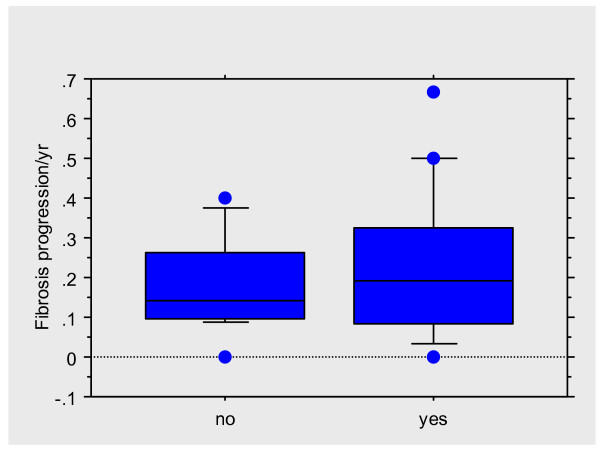
Box plot showing fibrosis progression rates/year in those with (yes) and without (no) hepatic steatosis.

Duration of stavudine (44.9 ± 21.5 vs. 24.8 ± 20.6) and didanosine (34.0 + 22.1 vs. 11.7 ± 10.4) were longer in those with fibrosis stage ≥ 3, though the differences did not achieve statistical significance (p value = 1.0 and 0.80 respectively). Factors that were significantly associated with fibrosis stage ≥ 3 on univariate analysis (p ≤ 0.1) (HIV diagnosed before or after 1996, cholestrol/HDL ratio, platelet count, grade of hepatic steatosis, necroinflammatory grade, and CD4 counts at time of the biopsy) were then entered into a step wise logistic regression model. The following were independently associated with advanced fibrosis: grade of necroinflammation (OR 1.9 (95% CI 1.1–3.3, p = 0.030), and grade of hepatic steatosis (OR 3.6 (95% CI 1.0–12.7), p = 0.045). There were 15 patients who had both a necroinflammatory grade ≥ 4 (mean grade in the whole group) and steatosis. Prevalence of cirrhosis in this group was 40%, compared to 13% in those without any of these two factors (p = 0.02). Fig 3 shows the fibrosis progression rates/year (FPR) in those with and without hepatic steatosis. The FPR was faster in the former though the differences did not achieve statistical significance, probably due to the small sample size.

Of the eight patients with more than one biopsy, the mean interval between the biopsies was 52 ± 17.9 months. Five had fibrosis progression ≥ one stage of whom 4 (80%) showed evidence of hepatic steatosis in the index biopsy.

## Discussion

This study has shown that 58% of HIV-HCV coinfected patients (of whom 72% were on ART) have evidence of hepatic steatosis on liver biopsy, and this is consistent with earlier data [9-11]. Those who had received an NRTI, either alone, or as part of sequential therapy were twice as likely to have steatosis (70%–82%) compared to those who had received HAART only therapy (41%). Independent predictors of hepatic steatosis were absence of HAART only therapy, and presence of cirrhosis. Neither PI or NRTI therapy was independently associated with steatosis. 45% of patients had advanced fibrosis and both grade of stetaosis and NI were independent predictors of advanced fibrosis. In the subgroup with > 1 biopsy, 80% of those with fibrosis progression had steatosis on the index biopsy.

The lowest prevalence of hepatic steatosis was observed in the HAART only group. Furthermore, the duration between HIV diagnosis and HAART initiation was longer for those with steatosis (47.4 vs. 40.4 months). This suggests that receiving HAART early, may in some way be protective against development of subsequent hepatic steatosis. Prior authors have also found a lower prevalence of hepatic steatosis with certain antiretroviral drugs (NNRTI) [1].

So how do we explain this negative association between HAART and hepatic steatosis? It may be partly related to the fact that NNRTIs (especially nevirapine) do not result in insulin resistance and may also be associated with elevations in high density lipoproteins (HDL) [17]. Secondly, HAART has been known to attenuate hepatic NI in coinfected patients [16,18] and there may in fact be an association between NI and hepatic steatosis. This could be because the HCV core protein leads to oxidative stress [19] and hepatic steatosis could result from the inflammation associated with this oxidative stress [20]. One could therefore speculate that appropriately initiated HAART could attenuate hepatic NI (either directly or via immune restoration induced changes in inflammatory cytokines) [21,22], which in turn may reduce hepatic steatosis.

Steatosis has been independently associated with advanced fibrosis in HCV monoinfected patients [6,23] though this remains controversial in HIV-HCV subjects [9-11]. In this study, those with advanced hepatic fibrosis had a significantly higher grade of hepatic steatosis compared to those with less severe hepatic fibrosis, (consistent with Sulkowski et al's data). In fact in our study 80% of patients with fibrosis progression had hepatic steatosis in the index biopsy. Also, a recent study has observed higher prevalence of hepatic steatosis in HIV-HCV coinfected patients compared with HCV monoinfected subjects (72.1% vs. 52%) [14]. Therefore one of the factors contributing to the accelerated fibrosis progression in HIV-HCV coinfected patients could be the increased prevalence of hepatic steatosis.

**Figure 4:**
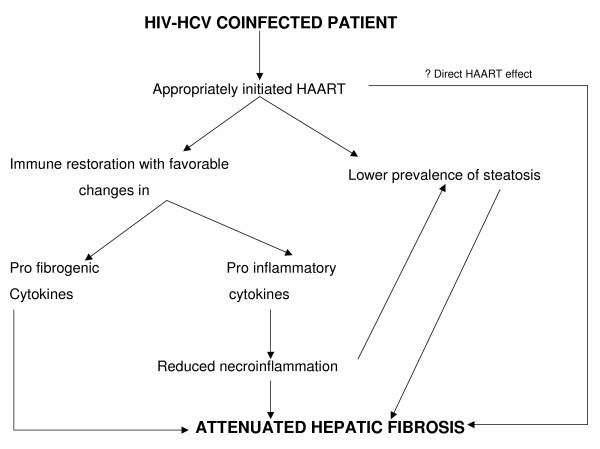
Proposed mechanism/s for attenuation of hepatic fibrosis observed with HAART in HIV-HCV coinfected subjects.

The effects of HAART on liver fibrosis in HIV-HCV coinfected patients remain controversial. Recent data however, do indicate that HAART may attenuate hepatic fibrosis in this cohort especially if patients receive HAART (rather than NRTI or sequential therapy) at onset, and if therapy is associated with successful viral suppression [16,24]. Data from this study further corroborates this hypothesis as 70% of those with fibrosis stage < 3 had their HIV diagnosed in or after 1996, indicating they were more likely to receive HAART at onset. The underlying mechanism/s responsible for this slower fibrosis progression associated with HAART remain speculative, but could be related to immune restoration induced changes in pro inflammatory and profibrogenic cytokines, a direct effect of HAART on liver fibrosis [21,25,26], or as indicated in this study, the relatively lower prevalence of steatosis (Fig 4).

We however accept the limitations of this study, including its retrospective design, and the small sample size of the coinfected cohort. In addition liver biopsies were performed in only carefully selected patients. Though this clearly resulted in a selection bias, such a bias is true for most studies in this area, and in our opinion is unavoidable.

In conclusion despite a low prevalence of obesity and DM, 58% of HIV-HCV coinfected patients have evidence of hepatic steatosis histologically. Absence of HAART only therapy was independently associated with steatosis in this study. The presence of hepatic steatosis was also associated with more advanced hepatic fibrosis in the coinfected cohort. The comparatively lower prevalence of hepatic steatosis seen in those that receive HAART only (rather than NRTIs only or sequential therapy) may contribute to the attenuated hepatic fibrosis that has been observed with this therapy [17,22].

## Competing interests

The authors declare that they have no competing interests.

## Authors' contributions

SV participated in the study design and was responsible for data collection, statistical analysis and drafting of the manuscript. RG performed the histological analysis and contributed to drafting of the manuscript. JM conceived of the study and contributed to drafting of the manuscript. All authors read and approved the final manuscript.
